# The prognostic significance of microsatellite instability in colorectal cancer: a Swedish multi-center study

**DOI:** 10.1007/s00384-023-04480-z

**Published:** 2023-07-17

**Authors:** Petri Rantanen, Anne Keränen, Shabane Barot, Sam Ghazi, Annelie Liljegren, Caroline Nordenvall, Annika Lindblom, Ulrik Lindforss

**Affiliations:** 1 Department of Molecular Medicine and Surgery, Karolinska Institutet, Stockholm, Sweden; 2 Department of Laboratory Medicine, Division of Pathology, Karolinska Institutet, Stockholm, Sweden; 3 Department of Clinical Science and Education, Södersjukhuset, Karolinska Institutet Stockholm, Sweden; 4 Department of Oncology-Pathology, Karolinska Institutet, Stockholm, Sweden; 5 Department of Clinical Genetics, Karolinska Institutet, Stockholm, Sweden

**Keywords:** Colorectal cancer, Prognosis, Mismatch repair, Microsatellite instability

## Abstract

**Purpose:**

About 10 to 15% of patients with sporadic colorectal cancer display mutations in DNA mismatch repair (MMR) genes shown as microsatellite instability (MSI). Previous reports of colorectal cancer (CRC) indicate a better prognosis for patients with MSI tumors compared to patients with microsatellite stable (MSS) tumors. In this study, our aim was to investigate whether MSI is an independent prognostic factor in CRC.

**Patients and methods:**

Patients with stage I–III colorectal cancer and subject to curative surgery during 2002–2006 in the Swedish low-risk colorectal cancer study group cohort were eligible for inclusion. Deficient MMR (dMMR) status was analyzed by immunohistochemistry (IHC) and/or by MSI testing with polymerase chain reaction (PCR). Prognostic follow-up and treatment data were retrieved from patient records. Statistical analyses to assess MSI-status and prognosis were done using logistic regression and survival analyses using the Kaplan-Meier method and Cox regression hazards models adjusted for age, sex, stage, comorbidity, and tumor location.

**Results:**

In total, 463 patients were included, MSI high tumors were present in 66 patients (14%), and the remaining 397 were MSS/MSI low. Within 6 years, distant recurrences were present in 9.1% and 20.2% (*P* = 0.049), and death occurred in 25.8% and 31.5% in MSI and MSS patients, respectively. There was no statistically significant difference in overall mortality (HR 0.80, 95% CI 0.46–1.38), relapse-free survival (HR 0.82, 95% CI 0.50–1.36), or cancer-specific mortality (HR 1.60, 95% CI 0.73–3.51).

**Conclusion:**

Despite distant metastases being less common in patients with MSI, there was no association between MSI and overall, relapse-free, or cancer-specific survival.

## Introduction

Inactivation of the DNA mismatch repair (MMR) system leads to an accumulation of mutations in the short repeating base pair units called microsatellites [1]. This accumulation of errors renders the microsatellite instability (MSI) phenotype, sometimes also referred to as the hypermutator phenotype [2]. In sporadic colorectal cancer (CRC), this is mainly due to epigenetic inactivation of the MMR genes, most commonly *MLH1*, while in the hereditary condition of Lynch’s syndrome (LS), it is caused by germline mutations [3–5]. LS attributes to about 3% of the CRC cases, and MSI is present in 10–15% of the sporadic cases of CRC [6–8]. Common clinical and pathological features of deficient mismatch repair (dMMR) CRCs include proximal tumor location, extensive tumor-infiltrating lymphocytes, higher T-stage, and poor differentiation [9]. The detection of MMR status and MSI is mainly done by immunohistochemistry (IHC) and PCR-based MSI testing, and the concordance between the methods is high and well documented [10–12].

When discussing the prognostic impact of MSI in CRC, one must also address the predictive value of MSI regarding chemotherapy, since a vast majority of studies come from different adjuvant trials. Several earlier studies suggested a better stage-adjusted survival for patients with MSI versus MSS tumors [13–17]. In a pooled analysis of stage II–III patients, MSI was associated with an improved disease free survival (DFS) in patients naïve for chemotherapy [18]. Non-interventional studies of the association between MSI and prognosis following chemotherapy are contradictive [18, 19]. The largest pooled study including patients from several different randomized 5-Fu based adjuvant treatment trials concluded that patients with MSI tumors had reduced recurrence rates, delayed time to relapse, and fewer distant recurrences [20]. Also, the rate of distant recurrences was reduced in MSI patients with stage III disease receiving adjuvant 5-Fu treatment implying a benefit for chemotherapy in MSI patients. It must be mentioned that only a subset of patients from these trials are represented in the study due to available tissue specimens. A meta-analysis of 3690 CRC patients of which 75% was stage III and investigated the predictive role of MSI in adjuvant chemotherapy treatment (mostly 5-Fu based) [21]. It showed that MSI patients that received adjuvant chemotherapy had a survival benefit compared to MSS patients. However, in analysis restricted to MSI patients, adjuvant chemotherapy was not associated with improved DFS or overall survival.

The risk of selection bias is evident in previously mentioned pooled studies since only patients with available tissue material have been eligible for inclusion. This means that patients only represent a subset of the overall study cohorts. Also, patients in many previous studies were drawn from several clinical trials conducted now 30 to 40 years ago [20].

So, despite large efforts to settle the score on the prognostic and predictive impact of MSI in CRC, the results are not concordant. In this study, we have investigated a well-defined Swedish population treated for CRC to further elucidate whether MSI could be considered an independent prognostic factor.

## Method

### Study population

**Fig. 1:**
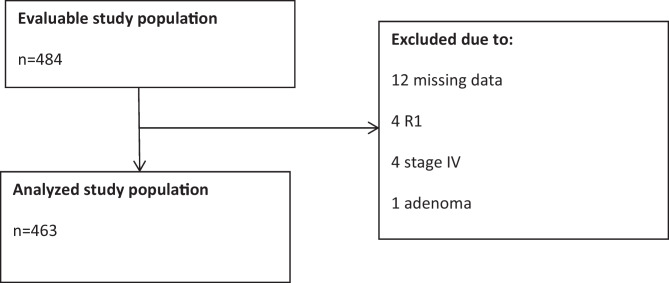
Flowchart

Patients were recruited from the Swedish low-risk colorectal cancer study group, which is a national multi-center collaborative including 14 different surgical clinics in the middle of Sweden [22]. From this cohort, a subset of 484 patients with CRC treated in the Stockholm area during the years 2002–2006 were evaluated, and inclusion criteria were curative surgery for stage I–III disease, radical surgery, known MMR-status, and available follow-up. After exclusion, a total of 463 patients remained for analysis (Fig. 1). All surgical specimens were evaluated by the same pathologist, and an extended detailed morphological description was made [23]. Informed consent was obtained from all patients and approval from the local Ethics Committee in Stockholm (2017/57-31/4).

Patient records were scrutinized for treatment and follow-up data according to protocol by two investigators (P.R. and S.B.). Post-operative complications and co-morbidity according to the American Society of Anesthesiologists (ASA) Physical Status Classification System were recorded.

The cohort includes 10 patients with Lynch syndrome. These patients have not been excluded since we assume that the phenotype of MSI is the determining factor for prognosis rather than how it is acquired. Due to this, a separate sensitivity analysis was conducted. A detailed description of the Lynch syndrome patients, including the pathogenic variants, is presented in a previous study [24].

### Exposure

The immunohistochemistry (IHC), MSI testing, and in part of the cases mutation screening for this cohort have been described in a previous study [24] and presented here briefly as follows.

For the IHC, analyses tissue from colorectal adenocarcinomas were used. Before analysis, the material was fixed in formalin and embedded in paraffin.

Four different monoclonal antibodies for MLH1, MSH2, MSH6, and PMS2 were used and staining done in accordance with instructions from the manufacturer.

Samples were assessed as MMR deficient if they demonstrated partial or total loss of nuclear expression in invasive cancer cells with synchronous remaining expression in normal cells, functioning as an internal positive control.

For MSI-testing, a commercial kit was used in accordance with instructions from the manufacturer. Tumor tissue embedded in paraffin was dissected before MSI-analysis. A multiplex PCR was used to amplify DNA in selected microsatellite markers, namely, BAT-26, NR-21, BAT-25, MONO-27, and NR-24. Tumors were reported as MSI-high when three or more of the five mononucleotide markers showed a pattern consistent with MSI. In addition, mutation screening was performed for part of the cases mainly using Sanger sequencing.

### Outcome

The patients were followed from date of surgery until event or six years of follow-up. The main outcomes were overall survival (OS), cancer-specific survival (CSS), and relapse-free survival (RFS). The outcome of interest in the OS analyses was death whereas date of migration or end of follow-up were considered censoring events. Similarly, date of CRC cancer death was the outcome of interest in the cancer-specific survival analyses and date of relapse or death in the relapse-free survival analyses.

### Statistics

Median and interquartile ranges are reported for continuous variables whereas frequencies and percentages are reported for categorical variables. The *P* values in descriptive tables come from Wilcoxon and Chi-squared tests for continuous and categorical variables, respectively.

Kaplan-Meier curves are compared for overall survival, recurrence free survival, and cancer-specific survival. Difference in the survival between groups of interest is tested with multiple tests, including one that accounts for crossing of survival curves.

Cox regression models are used to present unadjusted and adjusted hazard ratios for MMR-status and outcome. The adjusted analyses are adjusted for sex, age, cancer stage, comorbidity, and tumor location. *P* values are two sided, values below 5% are deemed significant, and 95% confidence intervals are used throughout. Proportional hazard tests were also conducted. There was no violation of the proportional hazard assumption for the analyses of overall and cancer-specific survival. However, for the analysis of relapse-free survival, there was a violation for age and stage. Hence, the multivariable model for relapse-free survival was stratified for age and stage within the Cox regression model. Analyses were performed in R version 4.1.2.

## Results

### Patient characteristics

A total of 463 stage I–III patients undergone R0-resection surgery were divided into two groups based on their MMR status. MSI tumors were present in 66 patients (14.2%), and the rest were MSS. The median ages were similar in the two groups (Table 1). In the MSI group, a significant predominance was seen for female gender (*P* = 0.016) as well as for right-sided tumor location (*P* < 0.001). Comorbidity according to the ASA classification did not differ.

**Table 1:** Preoperative patient demographics and clinical data in 463 patients undergoing surgery for primary colorectal cancer

		**MSS**		**MSI**		
		*N* = 397	%	*N* = 66	%	*P* value
**Age, median (range) years**		70.00 (62.00, 79.00)	85.7	71.00 (61.25, 78.75)	14.3	0.869
**Age interval**	< 60	74	18.6	16	24.2	0.478
	60–75	189	47.6	27	40.9	
	> 75	135	33.8	23	34.8	
**Sex**	Male	224	56.2	26	39.4	< 0.05
	Female	174	43.8	40	60.6	
**ASA grade**	1–2	310	77.8	54	81.8	0.571
	3–5	88	22.2	12	18.2	
**Tumor location**	Right	115	29.0	52	78.8	< 0.05
	Left	132	33.2	9	13.6	
	Rectum	150	37.8	5	7.6	
Rectal cancer patients only						
**Neoadjuvant radiotherapy**	No	46	30.7	1	20.0	0.987
	Yes	104	69.3	4	80.0	
**Concomitant** **chemo-radio**	No	143	95.3	5	100.0	1.000
	Yes	7	4.7	0	0.0	

### Disease and treatment characteristics

Treatment, complications, and histopathological data are presented in Table 2. There were no significant differences related to surgical or adjuvant treatment characteristics between the MSI and MSS groups. The average number of chemo cycles was 11 in the MSI group and 10 in the MSS group. Stage II was more common among MSI patients, but otherwise, there were no significant histopathological differences. Complications were evenly distributed between the groups (Table 3).

**Table 2:** Surgical and histopathological details in 463 patients undergoing surgery for primary colorectal cancer

		**MSS**		**MSI**		
		*n*	%	*n*	%	*P* value
**Operative setting**	Elective surgery	346	87.2	57	86.4	1.000
	Emergency surgery	51	12.8	9	13.6	
**Complications**	No	227	57.2	39	59.1	0.876
	Yes	170	42.8	27	40.9	
**Surgical complications**	Yes	89	22.4	9	13.6	0.146
**Other complications**	Yes	135	34.0	21	31.8	0.836
**Re-operation**	No	370	93.2	64	97.0	0.37
	Yes	27	6.8	2	3.0	
**Pathological T stage**	pT1-2	103	25.9	8	12.1	0.107
	pT3	253	63.7	53	80.3	
	pT4	38	9.6	5	7.6	
	pTx	2	0.5	0	0.0	
	Missing	1	0.3	0	0.0	
**Pathological N stage**	pN0	230	57.9	48	72.7	0.096
	pN1-2	153	38.5	18	27.3	
	pNX	12	3.0	0	0.0	
	Missing	2	0.5	0	0.0	
**Stage**	I	84	21.2	8	12.1	< 0.05
	II	159	40.1	40	60.6	
	III	154	38.8	18	27.3	
**Adjuvant chemotherapy**	No	318	80.1	51	77.3	0.716
	Yes	79	19.9	15	22.7	
**Adj. chemo** **regimen**						
**5-Fu**		49	12.3	8	12.1	0.699
**5-Fu combination**		30	7.6	7	10.6	
Rectal cancer patients only						
**Neoadjuvant** **radiotherapy dose**						
**25 Gy**		86	57.3	3	60.0	< 0.05
**50 Gy**		18	12.0	1	20.0	

### Outcomes

**Fig. 2:**
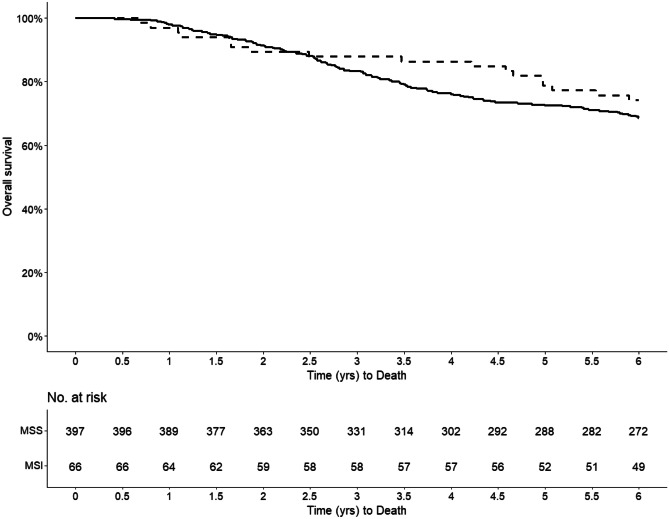
Kaplan-Meier curve illustrating the overall survival stratified by MSI status (MSI = dashed line; MSS = solid line)

**Fig. 3:**
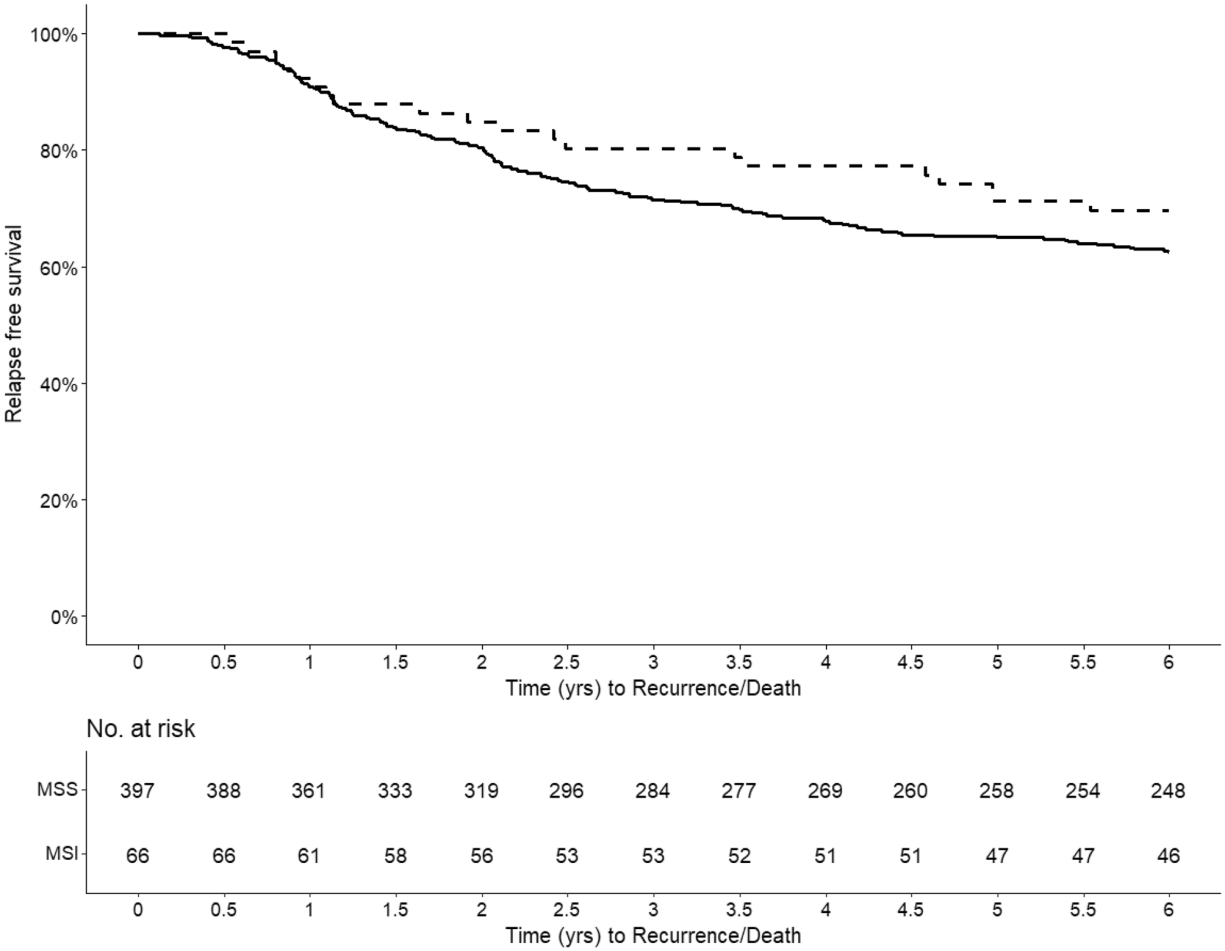
Kaplan-Meier curve illustrating the relapse-free survival stratified by MSI-status (MSI = dashed line; MSS = solid line)

**Fig. 4:**
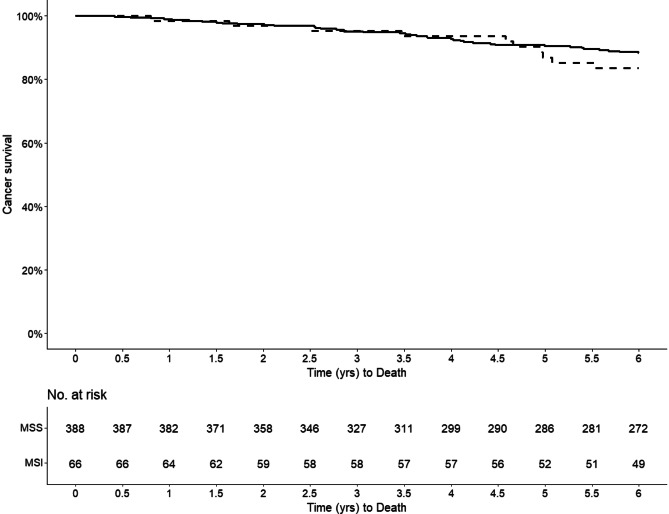
Kaplan-Meier curve illustrating the cancer-specific survival stratified by MSI status (MSI = dashed line; MSS = solid line)

There was no death within 30 or 90 days after surgery. Median follow-up time was 6 years. Within the first 6 years, 15.2% and 24.4% of the patients in the MSI and MSS groups, respectively, suffered relapse. Distant metastases were most common and present in 9.1% and 20.2% of the MSI and MSS patients, respectively (*P* = 0.049) (Table 3). The Kaplan-Meier curves revealed no significant difference in OS, RFS, or CSS between MSI and MSS. The 6-year OS were 74.2% and 68.5% (Fig. 2) for MSI and MSS patients, respectively, and the corresponding numbers for RFS were 69.7% and 62.5% (Fig. 3), and for CSS 74.2% and 70.1% (Fig. 4). There was no statistically significant difference in overall mortality (HR 0.80, 95% CI 0.46–1.38) or cancer-specific mortality (HR 1.60, 95% CI 0.73–3.51) or relapse-free survival (HR 0.82, 95% CI 0.50–1.36 (Table 4). Sensitivity analysis, where patients with Lynch’s syndrome (*n* = 10) were excluded, revealed similar results as the main analyses (data not shown).

**Table 3:** Six-year survival data in 463 patients undergoing surgery for primary colorectal cancer

		**MSS**		**MSI**		
		*n*	%	*n*	%	*P* value
**Postoperative mortality (within 30 days)**	No	397	100.0	66	100.0	NA
	Yes	0	0	0	0	
**Postoperative mortality** **(within 90 days)**	No	397	100.0	66	100.0	NA
	Yes	0	0	0	0	
**Total mortality**	No	272	68.5	49	74.2	0.429
	Yes	125	31.5	17	25.8	
**Recurrent disease**	No	300	75.6	56	84.8	0.134
	Yes	97	24.4	10	15.2	
**Local recurrence**	No	375	94.5	62	93.9	1.000
	Yes	22	5.5	4	6.1	
**Distant recurrence**	No	317	79.8	60	90.9	< 0.05
	Yes	80	20.2	6	9.1

**Table 4:** Multivariate Cox regression analysis of factors affecting 6-year mortality

		**Univariable**		**Model 1**^**a**^		**Model 2**^**b**^		**Model 3**^**c**^	
		HR	95% CI	HR^a^	95% CI	HR^b^	95% CI	HR^c^	95% CI
**Overall mortality**									
MSI status	MSS	Ref		Ref		Ref		Ref	
	MSI	0.78	0.47; 1.30	0.79	0.47; 1.32	0.77	0.45; 1.34	0.80	0.46; 1.38
**Relapse-free survival**									
MSI status	MSS	Ref		Ref		Ref		Ref	
	MSI	0.76	0.48; 1.21	0.76	0.47; 1.21	0.77	0.47; 1.27	0.82	0.50; 1.36
**Cancer-specific mortality**									
MSI status	MSS	Ref		Ref		Ref		Ref	
	MSI	1.40	0.70; 2.81	1.39	0.67; 2.90	1.61	0.73; 3.57	1.60	0.73; 3.51

^a^Adjusted for age, sex, ASA

^b^Additionally adjusted for tumor location

^c^Additionally adjusted for stage

## Discussion

In this well-defined cohort of patients with stage I–III colorectal cancer, there was no significant association with MSI status and prognosis.

Previous studies of prognosis and predictive value of MSI show somewhat conflicting results. A variety of different settings in terms of patient selection, study design, and heterogeneous cohorts may affect the results and contribute to the diverging results. In studies of MSI and prognosis, selection bias is a problem. To increase the number of patients, several pooled analyses have been performed originating from randomized trials. Pooled analysis can result in counterintuitive and spurious results if data is not weighted. In a systematic review, MSI was associated with improved overall survival in patients treated with surgery alone as well as with adjuvant 5-Fu [25], which was supported by a meta-analysis that showed an improved overall survival and disease-free survival in MSI patients [26]. However, systematic reviews and meta-analysis never get better than the included data, why large cohort studies with good internal validity are preferable.

Our results failed to demonstrate a prognostic advantage of MSI regarding relapse free survival, overall survival, or cancer-specific survival, which is in line with previous studies [27–29]. In comparison to other well-defined cohorts, one single-center study (*n* = 613) reported no association between MSI status and overall survival [30]. However, relapse-free survival was significantly improved in patients with MSI and sub-group analyses revealed that improved outcome applied to stage II patients. A slightly smaller study of patients with CRC stage II–III (*n* = 245), where most patients received adjuvant chemotherapy, showed no association between MSI status and overall survival or disease-free survival. A sub-analysis, that should be interpreted with caution, reported an impaired prognosis in stage II MSI patients compared with MSS patients [31].

In line with previous studies, distant metastases were less common in MSI compared with MSS patients [20, 32], but there was no significant difference in relapse-free survival between the two groups. Some propose that the prognostic impact of MSI is stage dependent and substantially stronger in stage II than in stage III [33]. Despite a fairly large and well-defined cohort, we still believe that sample size prevents a trustworthy subgroup analysis.

If the independent impact of MSI in CRC was evident, additional studies on the subject would be redundant and the matter settled already. A majority of studies point in the direction of MSI as a prognostically beneficial factor, and our study does not contradict that. It is speculated that the possible prognostic advantage of MSI is related to immunosurveillance. Many other parameters seem to affect prognosis more than MSI, but it is still one piece of the puzzle to be taken into consideration. Especially in right-sided cancers, MSI may be of considerable importance, since the proportion of MSI is higher than on the left side of colon and rectum. Also, it seems that MMR status is an important factor in relation to immunotherapy treatment such as checkpoint blockade, for which we can hope for therapeutic progress in the future [34].

The problem with many studies including ours might be that MSI itself could be due to heterogenous mechanisms where the evasion of immune cells is of severe importance. The high load of mutational neoantigens in dMMR/MSI-cancers leads to a pronounced anti-tumoral response such as infiltration of CD8T cells, an important reaction within MSI tumors [35]. The immunoselection during the development of the tumor, caused by possible mechanisms mentioned above, might be an answer to diverging results, which in turns requires even more distinctive subclassification of the MSI phenomenon. Antigen expression, or the loss of it, might explain why MSI in itself is not that easily classified as one group of patients with similar mechanisms and response to immunotherapy.

This is a large cohort of colorectal cancer patients with high quality data of patient and tumor characteristics and treatment. There was no loss to follow-up, and it could be presumed that all recurrences of clinical impact have been identified. In difference from the pooled studies, the study population in the current study has been systematically collected for evaluation and is not a mix from several different trials. Hence, the risk of selection bias is low. In addition, IHC/MSI testing has been performed in a homogenous manner decreasing the risk of information bias. The patients are included from several centers in the Stockholm area, and results are most likely generalizable to Swedish patients as well as other European countries.

However, despite a comparatively large cohort, the study is still limited by its size only including 66 patients with MSI, making it impossible to analyze the effect of MSI in different subgroups. In addition, the number of patients that received chemotherapy is limited. Therefore, we have not addressed the issue of MSI as a predictive factor for chemotherapy response.

## Conclusion

In this large cohort of CRC-patients, distant recurrences were less common in MSI-patients. Despite the difference in recurrences, MSI was not significantly associated with prognosis. Larger studies are needed to explore MSI as a prognostic marker, possibly in subgroups defining the effect of immune evasion, especially in the new era of immunotherapy.

## Data Availability

The datasets generated during and analyzed during the current study are available from the corresponding author on reasonable request.

## References

[1] Thibodeau SN, Bren G, Schaid D (1993). Microsatellite instability in cancer of the proximal colon. Science.

[2] Ionov Y (1993). Ubiquitous somatic mutations in simple repeated sequences reveal a new mechanism for colonic carcinogenesis. Nature.

[3] Aaltonen LA (1993). Clues to the pathogenesis of familial colorectal cancer. Science.

[4] Peltomäki P (1993). Microsatellite instability is associated with tumors that characterize the hereditary non-polyposis colorectal carcinoma syndrome1. Can Res.

[5] Poynter JN (2008). Molecular characterization of MSI-H colorectal cancer by MLHI promoter methylation, immunohistochemistry, and mismatch repair germline mutation screening. Cancer Epidemiol Biomark Prev.

[6] Hampel H (2008). Feasibility of screening for lynch syndrome among patients with colorectal cancer. J Clin Oncol.

[7] Ward R (2001). Microsatellite instability and the clinicopathological features of sporadic colorectal cancer. Gut.

[8] Vilar E, Gruber SB (2010). Microsatellite instability in colorectal cancer-the stable evidence. Nat Rev Clin Oncol.

[9] Jass JR (1998). Morphology of sporadic colorectal cancer with DNA replication errors. Gut.

[10] Cheah PL (2019). Screening for microsatellite instability in colorectal carcinoma: practical utility of immunohistochemistry and PCR with fragment analysis in a diagnostic histopathology setting. Malays J Pathol.

[11] Hissong E (2018). Assessing colorectal cancer mismatch repair status in the modern era: a survey of current practices and re-evaluation of the role of microsatellite instability testing. Mod Pathol.

[12] Shia J (2008) Immunohistochemistry versus microsatellite instability testing for screening colorectal cancer patients at risk for hereditary nonpolyposis colorectal cancer syndrome. Part I. The utility of immunohistochemistry. J Mol Diag 10(4): p. 293–300 10.2353/jmoldx.2008.080031 PMC2438196 18556767

[13] Sinicrope FA (2006). Prognostic impact of microsatellite instability and DNA ploidy in human colon carcinoma patients. Gastroenterology.

[14] Gafà R (2000). Sporadic colorectal adenocarcinomas with high-frequency microsatellite instability. Cancer.

[15] Halling KC et al (1999) Microsatellite instability and 8p allelic imbalance in stage B2 and C colorectal cancers. J Natl Cancer Inst 91(15): p. 1295–1303 10.1093/jnci/91.15.1295 10433618

[16] Lanza G (2006). Immunohistochemical test for MLH1 and MSH2 expression predicts clinical outcome in stage II and III colorectal cancer patients. J Clin Oncol.

[17] Sargent DJ et al (2014) Prognostic impact of deficient mismatch repair (dMMR) in 7,803 stage II/III colon cancer (CC) patients (pts): a pooled individual pt data analysis of 17 adjuvant trials in the ACCENT database. J Clinical Oncol 32(15_suppl): p. 3507–3507

[18] Sargent DJ (2010). Defective mismatch repair as a predictive marker for lack of efficacy of fluorouracil-based adjuvant therapy in colon cancer. J Clin Oncol.

[19] Tejpar S et al (2009) Microsatellite instability (MSI) in stage II and III colon cancer treated with 5FU-LV or 5FU-LV and irinotecan (PETACC 3-EORTC 40993-SAKK 60/00 trial). J Clin Oncol 27(15_suppl): p. 4001–4001

[20] Sinicrope FA (2011). DNA mismatch repair status and colon cancer recurrence and survival in clinical trials of 5-fluorouracil-based adjuvant therapy. J Natl Cancer Inst.

[21] Des Guetz G (2009). Does microsatellite instability predict the efficacy of adjuvant chemotherapy in colorectal cancer? A systematic review with meta-analysis. Eur J Cancer.

[22] Forsberg A (2017). Defining new colorectal cancer syndromes in a population-based cohort of the disease. Anticancer Res.

[23] Ghazi S (2010). Colorectal cancer susceptibility loci in a population-based study. Am J Pathol.

[24] Keränen A (2018). Testing strategies to reduce morbidity and mortality from Lynch syndrome. Scand J Gastroenterol.

[25] Popat S, Hubner R, Houlston RS (2005). Systematic review of microsatellite instability and colorectal cancer prognosis. J Clin Oncol.

[26] Guastadisegni C (2010). Microsatellite instability as a marker of prognosis and response to therapy: a meta-analysis of colorectal cancer survival data. Eur J Cancer.

[27] Kim GP (2007). Prognostic and predictive roles of high-degree microsatellite instability in colon cancer: a national cancer institute–national surgical adjuvant breast and bowel project collaborative study. J Clin Oncol.

[28] Lamberti C (2007). Microsatellite instability did not predict individual survival of unselected patients with colorectal cancer. Int J Colorectal Dis.

[29] Salahshor S (1999). Microsatellite instability in sporadic colorectal cancer is not an independent prognostic factor. Br J Cancer.

[30] Merok MA (2013). Microsatellite instability has a positive prognostic impact on stage II colorectal cancer after complete resection: results from a large, consecutive Norwegian series. Ann Oncol.

[31] Shin US (2014). Is microsatellite instability really a good prognostic factor of colorectal cancer?. Ann Coloproctol.

[32] Hutchins G (2011). Value of mismatch repair, KRAS, and BRAF mutations in predicting recurrence and benefits from chemotherapy in colorectal cancer. J Clin Oncol.

[33] Roth AD et al (2009) Stage-specific prognostic value of molecular markers in colon cancer: results of the translational study on the PETACC 3-EORTC 40993-SAKK 60–00 trial. J Clin Oncol 27(15_suppl): p. 4002–4002 10.1200/JCO.2009.23.3452 20008640

[34] Le DT (2017). Mismatch repair deficiency predicts response of solid tumors to PD-1 blockade. Science.

[35] Buckowitz A (2005). Microsatellite instability in colorectal cancer is associated with local lymphocyte infiltration and low frequency of distant metastases. Br J Cancer.

